# Paternal Incarceration and Adolescent Well-Being: Life Course Contingencies and Other Moderators

**Published:** 2015

**Authors:** Raymond R. Swisher, Unique R. Shaw-Smith

## Abstract

Parental incarceration has been found to be associated with a wide range of negative outcomes in both childhood and adolescence. This Article uses data from the National Longitudinal Study of Adolescent Health (Add Health) to focus on the conditions under which associations of paternal incarceration with adolescent delinquency and depression are strongest. Paternal incarceration is most consistently and positively associated with adolescent delinquency. Associations of paternal incarceration with adolescent depression are weaker and more contingent on gender and other moderating factors. One important moderator is the respondent's retrospective reports that he or she was physically or sexually abused by a parent or other adult caregiver during childhood. For example, in the absence of sexual abuse, paternal incarceration is associated with higher depression among girls. When coupled with reports of sexual abuse, in contrast, paternal incarceration is not associated with girls' depression, suggesting a potential protective effect. The child having ever coresided with his or her father is also found to moderate associations, with paternal incarceration most strongly associated with delinquency and depression among girls who had ever coresided with their fathers. Examination of the duration and timing of paternal incarceration also pointed to gender differences.

## Introduction^1^

The dramatic increase in incarceration rates in the United States, which many have described as representing an era of “mass incarceration,” and its disproportionate impact on persons of lower socioeconomic and minority status, is now fairly well documented.^2^ In addition to recognizing the many disadvantages that follow incarceration for those imprisoned,^3^ concern is growing about the “collateral consequences” of mass incarceration for the children and families of incarcerated fathers and mothers.^4^

A first generation of studies revealed that parental incarceration was associated with a wide range of negative outcomes in childhood and adolescence, including internalizing (e.g., depression) and externalizing behavior (e.g., delinquency, violence),^5^ as well as educational outcomes.^6^ A second generation of research has raised important methodological and substantive issues, such as whether these associations are truly causal in nature, and has delved into the social mechanisms that account for the relationship between parental incarceration and youth outcomes.^7^ While the emerging consensus is that parental incarceration likely affects child and adolescent well-being, important research questions remain, including how associations vary across subgroups of the populations (e.g., boys versus girls) and how they are moderated by a variety of factors, including the parent–child relationship history and the timing, duration, and frequency of parental incarcerations.

The present study focuses on this last set of questions regarding the conditions under which parental incarceration is more or less detrimental. These are not simply knobby or trivial methodological issues. Rather, they touch on important debates within the research literature and policy debates. For example, researchers are increasingly asking whether there are circumstances under which parental incarceration may be protective or beneficial to youth, such as when family life is threatened by violence or abuse.^8^ Researchers from a human development or life course perspective paternal incarceration is associated with higher rates of are asking whether the impact of parental incarceration varies based on the stage in the life course in which it occurs (e.g., during very young childhood versus later in childhood or adolescence).^9^ Similarly, one might wonder whether it is more detrimental for a child if the parent is incarcerated once for less than a year (suggesting an isolated minor event) or multiple times throughout childhood and adolescence. Lastly, theory and research on gender differences and inequalities suggest that the effects of parental incarceration may vary across girls and boys.^10^ Focusing on paternal incarceration and its associations with adolescent delinquency and depression, we examine these questions and issues using data from the Add Health survey.

## I. Literature Review

### A. Parental Incarceration and Childhood Well-Being

In a review of the recent literature, Murray and Farrington conclude that parental incarceration is a risk factor for a wide range of negative outcomes during childhood, from externalizing behaviors such as aggression, violence, and crime, to internalizing outcomes such as depression, anxiety, and other mental health problems.^11^ There are a variety of mechanisms through which these associations likely operate, including loss of attachment due to separation from the parent, diminished familial financial resources, family conflict and instability, parental stress and ineffective parenting by the unincarcerated parent, as well as stigma, negative labeling, and social exclusionary processes.^12^

As these studies are based on observational samples and not experimental designs (for obvious reasons), scholars have rightly questioned whether selection effects or unobserved factors are driving the relationship between parental incarceration and youth outcomes. Research has shown that disadvantaged families and parents face a multitude of risk factors, including alcohol and drug use, domestic violence, family instability, economic uncertainty, and ineffective parenting that are likely associated with both parental incarceration and negative outcomes for youth.^13^ Therefore, parental incarceration may represent little additional risk to youth who reside in such tumultuous home environments. In extreme cases, such as if an incarcerated parent was abusive or exposed the child to dangerous situations, incarceration may even represent a relief from preexisting stressful circumstances.

Recent studies have used a variety of sophisticated statistical techniques to more fully address the selection issue. For example, Wildeman used a combination of fixed effects and propensity score models to more convincingly argue that paternal incarceration has a causal effect on childhood aggression among Fragile Families respondents.^14^ Geller and colleagues observed a similar association within the Fragile Families data using fixed effects and placebo regressions.^15^ Using propensity score models and data from the Pittsburgh Youth Study, Murray and colleagues similarly reported a significant positive association between parental incarceration and child behavioral problems.^16^ Thus the consensus emerging is that parental incarceration likely has a causal effect on child aggression and externalizing behavior.

The case is less clear regarding the effects of parental incarceration on child internalizing outcomes such as depression or anxiety. For example, Geller and colleagues found no association between paternal incarceration and child internalizing behavior at age five in the Fragile Families data.^17^ Based on a meta-analysis of some forty studies, Murray and colleagues concluded that there is no association between parental incarceration and child mental health problems or drug use.^18^ In contrast, Wakefield and Wildeman found evidence of a positive association between parental incarceration and child internalizing problems such as depression and anxiety, particularly among girls.^19^

### B. Parental Incarceration and Adolescent Well-Being

Parental incarceration is expected to have negative consequences in adolescence, in part because many of the same mechanisms influencing child well-being—such as the trauma of parental separation, family instability, economic strain, and negative stigma and labeling—would be expected to also undermine adolescent well-being. At the same time, the relative roles of these mechanisms are likely changing, and new influences are emerging due to the unique challenges of adolescence, like the growing influence of peers relative to parents, new romantic and sexual relationships, identity explorations, and the heightened anxiety associated with academic performance and its consequences for future socioeconomic attainments.^20^

The best evidence to date suggests that parental incarceration is a strong risk factor for delinquency and other problem behavior in adolescence. This association has been observed both in the United States and in international samples. For example, using data from the Cambridge Study in Delinquent Behavior (CSDB), Murray and Farrington found that separations due to parental incarceration were positively associated with a range of antisocial behaviors at ages fourteen, eighteen, and thirty-two, controlling for parental convictions, and compared to separations for other reasons (e.g., the death of parent or divorce).^21^ Murray and colleagues also found parental incarceration to be significantly associated with theft and, to a lesser degree, marijuana use, using data from the Pittsburgh Youth Study.^22^ Using data from the Project on Human Development in Chicago Neighborhoods (PHDCN), Wakefield and Wildeman found adolescents with an incarcerated parent to have higher delinquency, aggression, and externalizing behavior than youth without an incarcerated parent.^23^ Finally, using data from the Add Health study, researchers found paternal incarceration to be positively associated with serious delinquency and arrest,^24^ and marijuana and hard drug use,^25^ in both adolescence and the transition to adulthood.

As is true for studies of children, results are more mixed with respect to associations of parental incarceration with adolescent depression and other internalizing outcomes. In an analysis based on the CSDB, Murray and Farrington observed long-lasting effects of parental incarceration on boys' internalizing outcomes from ages fourteen all the way to age forty-eight.^26^ Similarly, research based on the PHDCN found parental incarceration to be associated with higher internalizing symptoms.^27^ Swisher and Roettger also reported a positive association between paternal incarceration and depression in adolescence and the transition to adulthood in the Add Health sample.^28^ On the other hand, Murray and colleagues found no association between parental incarceration and depression in the Pittsburgh Youth Study.^29^ Using a patient-based sample, Phillips and colleagues found that adolescents with incarcerated parents were more likely to present with attention deficit/hyperactivity and conduct disorders but were less likely to be diagnosed with major depressive symptoms.^30^ Moreover, based on their meta-analysis of forty studies, Murray and colleagues concluded that there was little evidence to suggest more than a very small association between parental incarceration and internalizing outcomes in childhood or adolescence.^31^

### C. Moderation by Abuse, Coresidence, Temporality, and Gender

Despite this seeming consensus regarding the effects on adolescent externalizing behaviors and a weaker-to-nonexistent association with internalizing outcomes, many important research questions remain unanswered. In particular, we still know relatively little about the conditions under which parental incarceration is most detrimental to youth outcomes. Stated alternatively, we need to know more about how associations between parental incarceration and child well-being are moderated by factors like the child's residential history with the incarcerated parent; family functioning prior to incarceration; the timing, duration and frequency of parental incarceration; and the gender of the child.

Take, for example, the issue of preexisting risk factors in the lives of youth with incarcerated parents, such as domestic violence. Though the incarceration of a parent is likely to always create tumult, incarcerations of an abusive or violent parent might be expected to provide some relief to the child and family. Wildeman examined this issue using Fragile Families data and observed that maternal reports of abuse by fathers lessened the effect of paternal incarceration on boys' physical aggression.^32^ Also consistent with a potentially protective effect was the finding that boys whose fathers were incarcerated for violent offenses were not more aggressive than other children, whereas those whose fathers were incarcerated for nonviolent offenses were significantly more aggressive comparatively.^33^ Geller also examined this issue using the Fragile Families data, but found no statistically significant difference when comparing those with and without abusive fathers in the effect of paternal incarceration on childhood aggression.^34^ Paternal incarceration was positively associated with behavioral problems for both groups.^35^ We extend examination of this issue to consider how instances of domestic physical and sexual abuse as experienced and reported by the child—as opposed to the mother— correlate with the outcomes of delinquency and depression in adolescence.

In addition to expecting less of an effect for youth whose fathers were abusive or violent, one might also predict that incarcerations of nonresident fathers would be less stressful than those of fathers with whom the child was living. This is an important question, as past research suggests that fewer than half of incarcerated fathers were living with a minor child in the month before they were incarcerated.^36^ Geller and colleagues considered this issue with respect to outcomes in childhood. They found that the association with aggression was stronger for children who were living with the father prior to incarceration, but that paternal incarceration was also associated with higher aggression for children not living with their fathers.^37^ We will consider how associations between paternal incarceration and adolescent delinquency and depression are moderated by having ever lived with the biological father.

When studying the potential consequences of a stressful life event such as parental incarceration, the life course perspective directs attention to issues of temporality, such as the timing or stage in the life course during which it occurs, and the duration of the life event. For example, does it matter if a parent was incarcerated before his or her child was even born? Does a parent being incarcerated during childhood have an effect on outcomes in adolescence, or are only more recent incarcerations of consequence? Yet these issues of the timing, duration, and frequency of parental incarcerations are understudied to date. One exception is Murray and colleagues' study of the relationship between parental incarceration and youth theft. The authors found that theft rates were not moderated by the timing of parental incarcerations occurring during young childhood, childhood, and adolescence.^38^ Another exception is work by Cho, which examined how associations between maternal incarceration and high school dropout rates varied by timing. Cho found boys were more responsive to timing than girls, with maternal incarcerations in early adolescence found to be most detrimental.^39^

Also not well understood is whether longer durations of parental incarceration are more detrimental than shorter durations, or whether a single incarceration is as consequential as repeated instances of incarceration.^40^ From a dose–response point of view, examining the role of frequency and duration may contribute to a better understanding of the causal nature of parental incarceration. For example, linear relationships between the frequency and duration of parental incarceration and the outcomes would likely strengthen the case for a causal relationship. Unusual patterns of association between the frequency and duration of incarceration may also shed some light on the plausibility of various mechanisms through which parental incarceration affects adolescent outcomes. Few studies have examined these issues. One exception is Cho's research on maternal incarceration and children dropping out of high school. Cho found boys to be more influenced by the frequency of maternal incarcerations, with girls more responsive to their length.^41^

Cho's research also raises the child's gender as a potential moderating factor. There are a number of reasons to suspect that boys and girls might respond differently to having an incarcerated father. Most obvious is that girls are of a different gender than their incarcerated fathers and thus may not identify with and interact with their fathers to the same degree as boys do; alternatively, they may look to their fathers for different things.^42^ For example, some research suggests that fathers are more likely to invest in the family when they have boys.^43^ On the other hand, some studies suggest father involvement is equally beneficial for boys and girls.^44^

In addition to Cho's research, several other studies have considered such gender differences. For example, Murray and colleagues' meta-analysis reported stronger associations between parental incarceration and antisocial behavior for boys.^45^ Research based on the Fragile Families project also observed stronger associations between paternal incarceration and aggression in childhood among boys.^46^ The present analysis will consider gender differences in the associations between paternal incarceration and adolescent depression by stratifying models by gender.

## II. Methods

### A. Sample

This study uses data from the National Longitudinal Study of Adolescent Health (Add Health).^47^ The Add Health study began in 1995 and sampled students in grades seven to twelve, their parents, and school administrators from one hundred thirty-two randomly selected schools in the United States. The original in-school sample contains approximately 90,000 students, out of which a subset of 20,745 students and, in most cases, one of their parents, was randomly selected for in-home interviews at Wave I. At Wave I, respondents were between the ages of eleven and twenty-one years old. Wave IV was obtained in 2007–2008 when the sample subjects ranged from ages twenty-four to thirty-two years old and had an 80% response rate.

The analytic sample is limited to respondents who participated in Waves I and IV and who have valid longitudinal sample weights (n = 14,800). Outcomes are taken from Wave I. Wave IV participation is required, as that is when respondents were asked retrospective questions about paternal incarceration. List-wise deletion of respondents with a very small percentage of missing data on key independent variables such as parental incarceration, parental education, and the outcomes, reduced the main analytic sample to 14,579 respondents.^48^

### B. Dependent Variables

#### Serious delinquency

Serious delinquency was measured using a scale of twelve items tapping self reports of aggressive behavior during the past twelve months, including: serious physical fighting resulting in injuries requiring medical treatment; using a weapon to get something; group fighting; shooting or stabbing someone; deliberately damaging property; pulling a knife or gun on someone; stealing something worth less than $50; stealing something worth more than $50; breaking and entering; selling drugs; and holding stolen property. Cronbach's alpha was 0.81 at Wave I and 0.79 at Wave II. Due to the skewed nature of the variable, we take its natural log.

#### Depression

Depression was assessed with a scale based on five questions assessing respondents' frequencies of emotions during the past week, including: (1) being satisfied with life; (2) feeling depressed; (3) being unable to shake off the blues; (4) being happy; and (5) feeling sad. Cronbach's alpha was 0.80 at Wave I and 0.79 at Wave II. Due to the skewed nature of depression, we also take its natural log.

### C. Independent Variables

#### Paternal incarceration

Paternal incarceration was measured by respondents' retrospective reports at Wave IV. Respondents were first asked, “Has your biological father ever spent time in jail or prison?” If they answered “yes,” they were asked, “How old were you when your biological father went to jail or prison (the first time)?” and, “How old were you when your biological father was released from jail or prison most recently?” From these questions, a set of mutually exclusive timing categories were created, including biological father first incarcerated: (1) before birth; (2) between birth and age five; (3) between ages six and eleven; (4) between ages twelve and age at Wave I; and (5) first incarcerations that occurred after Wave I. Incarcerations first occurring before birth are further distinguished by whether the last release also occurred prior to or after birth. A similar set of questions was asked about biological mothers, which we use in additional sensitivity analyses.

#### Duration Variable

We use questions about ages of first incarceration and last release to create a duration variable, which is broken into categories of: (1) less than one year; (2) two to three years; (3) four to five years; (4) six to nine years; and (5) ten or more years. A limitation of this measure is that we know nothing about what happened between the first incarceration and last release. Thus, long durations may indicate a single incarceration of a long duration or multiple shorter duration incarcerations experienced over a long period of time. For this reason, we discuss duration in terms of a respondent's duration of experience with paternal incarceration.

#### Incarceration Frequency

Respondents were also asked how many times their biological father had been incarcerated. Responses are aggregated into categories of: (1) once; (2) two or three times; and (3) four times or more. We code respondents reporting no knowledge of their paternal incarceration into a separate categorical variable and retained in the analysis. We code those that refused to answer questions regarding paternal incarceration as missing.

#### Child Gender

Gender was coded as girl = 1, and boy = 0. Respondents were not given the option to make alternative gendered identifications beyond this gender binary.

#### Child Age

Age is based on the respondent's calculated age at Wave I.

#### Race/Ethnicity

Race and ethnicity were assessed by self-reports of primary racial identification and Hispanic origin. Mutually exclusive categories of non-Hispanic White, Black, Asian, and other race were constructed, as well as an indicator for those of Hispanic background of any race.

#### Household Structure

The structure of the child's household at Wave I was classified into categories representing adolescents: (1) living with both biological parents; (2) living with one biological parent and a stepparent; (3) living with a single mother; (4) living with a single father; (5) living in some other family type; and (6) living alone. An indicator variable for whether the respondent had ever lived with their biological mother and father is used in some of the moderator analyses.

#### Physical abuse

Our classification of physical abuse is based on responses to the question: “Before your eighteenth birthday, how often did a parent or adult caregiver hit you with a fist, kick you, or throw you down on the floor, into a wall, or down stairs?” Response categories ranged from “this has never happened” to “more than ten times.” We aggregate responses into two categories: (0) never happened or one time and (1) happened more than one time.

#### Sexual abuse

Our classification of sexual abuse is based on respondents' answers to the question: “How often did a parent or other adult caregiver touch you in a sexual way, force you to touch him or her in a sexual way, or force you to have sexual relations?” Responses are again dichotomized as: (0) never happened and (1) happened one or more times. Sensitivity analyses based on alternative collapsing strategies resulted in a similar pattern of results.

### D. Analytic Strategy

As noted, all models are weighted by the Add Health project's longitudinal sample weights to adjust for varying probabilities of initial sampling and longitudinal retention. Multivariate ordinary least squares regression models are used to assess the relationship between biological paternal incarceration and the outcomes of delinquency and depression. Examination of how the timing, duration, and frequency of fathers' incarcerations are associated with the outcomes is assessed using a series of mutually exclusive categorical variables. Analyses of moderation by physical and sexual abuse in the family and by coresidence with the biological father are accomplished using interactions between these statuses and a single variable representing paternal incarcerations between birth and Wave I. Given our interest in gender differences, models are run for the full sample and are also stratified by gender, with results presented separately for boys and girls.

## III. Results

### A. Descriptive Statistics

Table 1 presents means and proportions for the full analytic sample, as well as comparisons of adolescents with and without an incarcerated father. Overall, 14.1% report that their father^49^ was at any time incarcerated. Column 2 presents descriptive statistics for respondents who reported that their father had ever been incarcerated, whereas all other respondents are in Column 3. In terms of the timing of paternal incarceration, 9.0% of respondents reported that he was first incarcerated before birth (combining those released before or after birth), compared to 27.0% between birth and age five, 46.0% between ages six and twelve, 5.9% during adolescence itself, and 14.6% at later ages. Turning to the number of times a father was incarcerated, 42.3% of respondents report a single incarceration, 20.7% report two or three incarcerations, and another 15.0% report four or more incarcerations. Twenty-two percent of respondents with an incarcerated father say they do not know how many times their father had been incarcerated. Rather than treat this portion of the sample as missing, we retain those who do not know the timing in the analysis as a separate category of interest in itself. Like children who never lived with their fathers, those who do not know how many times their fathers were incarcerated may have less connection to their fathers and hence be less influenced by their incarcerations.

Comparing those respondents whose fathers have or have not been incarcerated reveals several differences that are important for present purposes. First, notice that 29.6% of those with an incarcerated father report instances of repeated physical abuse or sexual abuse in the family during childhood, compared to 13.6% of those without an incarcerated father. Also, 83.0% of those with an incarcerated father had lived with their father at some time prior to Wave I, compared to 90.5% of other youth. Youth with an incarcerated father were also much less likely to be living with both biological parents at Wave I (29.0% compared to 60.5%), and considerably more likely to live with a single mother (32.7% versus 17.7%) or other family members (9.5% versus 4.8%). In terms of the outcome variables, we note that youth with an incarcerated father have higher logged delinquency (1.44 versus 1.20) and depression (0.36 versus 0.32) scores than do youth without an incarcerated father.

### B. Timing of Paternal Incarceration

We examine in our multivariate analyses whether these differences in delinquency and depression remain when controlling for family background and other characteristics, while also examining issues of timing, frequency, duration, and relationship histories. First, we examine variation in associations by the age of the child at the time that the father was first incarcerated.

In Table 2, categories of paternal incarceration timing are regressed on logged delinquency and logged depression scores. Due to logging, one can interpret the regression coefficients as a one-unit change in the independent variable that is associated with a (100 * b) percent change in the outcome. The left-hand set of columns present results for delinquency, with the right-hand set of columns representing models for depression. Within each set of models, Column 1 contains results for the full sample, whereas Columns 2 and 3 present models for boys and girls, respectively.

Beginning with delinquency, the first model shows that paternal incarcerations at any age prior to Wave I are associated with higher delinquency scores. The largest coefficient is for youth whose fathers were incarcerated before their birth but last released after their birth. For these youth, a father's incarceration is associated with 47.7% higher delinquency scores. First incarcerations between birth and age five, ages six to twelve, and in adolescence prior to Wave I are all significantly associated with higher delinquency. Incarcerations later in life are not associated with delinquency at Wave I. One might argue that these should not be included, since they occurred after the dependent variable. However, this type of variable is sometimes used in incarceration research as what is called a placebo regression or falsification test, as a variable that should not be associated with the outcome.^50^ If it is, it may suggest the influence of unobserved factors. A quick review of models for boys and girls in Columns 2 and 3 indicate a similar pattern of associations for both groups.

In the second set of depression models, a weaker association with paternal incarceration is observed. In fact, none of the timing variables are significantly associated with depression within the full sample. For males, only the placebo variable of paternal incarceration after Wave I is statistically significant. Among females, paternal incarcerations between ages six and twelve are significantly associated with a 3.5% higher incidence of depression. However, we note that a larger association is observed for the placebo variable, suggesting that unobserved factors may be driving the association for girls as well.

### C. Duration and Frequency of Paternal Incarceration

We next examine the role of the duration and frequency of paternal incarcerations. The top panel of Table 3 presents models that distinguish the duration of experience with paternal incarceration based on the questions of when the father was first incarcerated and last released. Though the table only presents coefficients for the focal variables, the models contain all control variables from the previous models including age, gender, race and ethnicity, family structure, and parent's education.

For the sample as a whole, it is observed that longer durations of experience with an incarcerated father are most strongly associated with delinquency. For example, respondents reporting a ten-year-or-longer period between first incarceration and last release have 36.7% higher delinquency than do youth without an incarcerated parent. At the same time, short experiences with incarceration of up to one year in length remain significantly associated with a 15.1% higher level of delinquency. Also note, however, that respondents who said they did not know the ages at which their fathers were incarcerated have nearly the same delinquency as those with the longest durations (i.e., 34.4% higher than youth without a parental incarceration). It is also noteworthy that the small number of youth who reported that their parent was still in prison at Wave IV have significantly lower delinquency than youth without an incarcerated father. Patterns of association between duration and delinquency are largely similar for boys and girls, though we note that only girls experience the delinquency suppressing effect of the father still being incarcerated at Wave IV.

A different pattern of relationships is observed between duration of experience with paternal incarceration and depression, in part owing to the overall weaker associations. For the sample as a whole, both short durations of up to a year and durations of ten years or more are associated with higher depression. Examination of the models by gender, however, reveals that this overall pattern represents two different gender-specific associations. Among boys, it is shorter durations of up to a year that are significantly associated with 4.1% higher depression than youth without an incarcerated father. The sign of the coefficients for longer durations is actually negative for boys, though not statistically significant. For girls, in contrast, it is the longer duration experiences with paternal incarceration that are more strongly associated with depression.

The bottom panel in Table 3 presents model results examining the role of frequency or the number of times that a father was incarcerated. As with duration, frequency of paternal incarceration exhibits a dose-response pattern, with higher frequencies of incarceration associated with higher delinquency, both in the full sample and for boys and girls. The pattern is most pronounced for girls. For example, regression coefficients increase steadily from 0.14, to 0.22, to 0.48 (all statistically significant), for one time, two or three times, and four or more incarcerations, respectively. Not knowing the frequency of paternal incarceration is also associated with higher levels of delinquency.

As was true for duration, a gender-specific pattern of associations is observed between frequency and depression. Among boys, it is only one-time paternal incarcerations that are significantly associated with depression. For girls, in contrast, it is multiple incarcerations of the father that are significantly associated with depression. We also note that among girls, not knowing how many times the father was incarcerated is associated with the highest depression (i.e., 7.1% higher depression than for girls without an incarcerated father). We save interpretation of these gender differences for the discussion section.

### D. Histories of Physical and Sexual Abuse

We next turn our attention to the moderating role of relationship histories. In the top panel, logged delinquency and depression are regressed on paternal incarceration, reports of physical abuse in the family during childhood, and interactions between paternal incarceration and abuse. A similar set of models examining interactions of paternal incarceration with reports of sexual abuse are presented in the bottom panel. To simplify the interactions, we aggregate all fathers' incarcerations that took place between the respondent's birth and Wave I.

**Figure 1:**
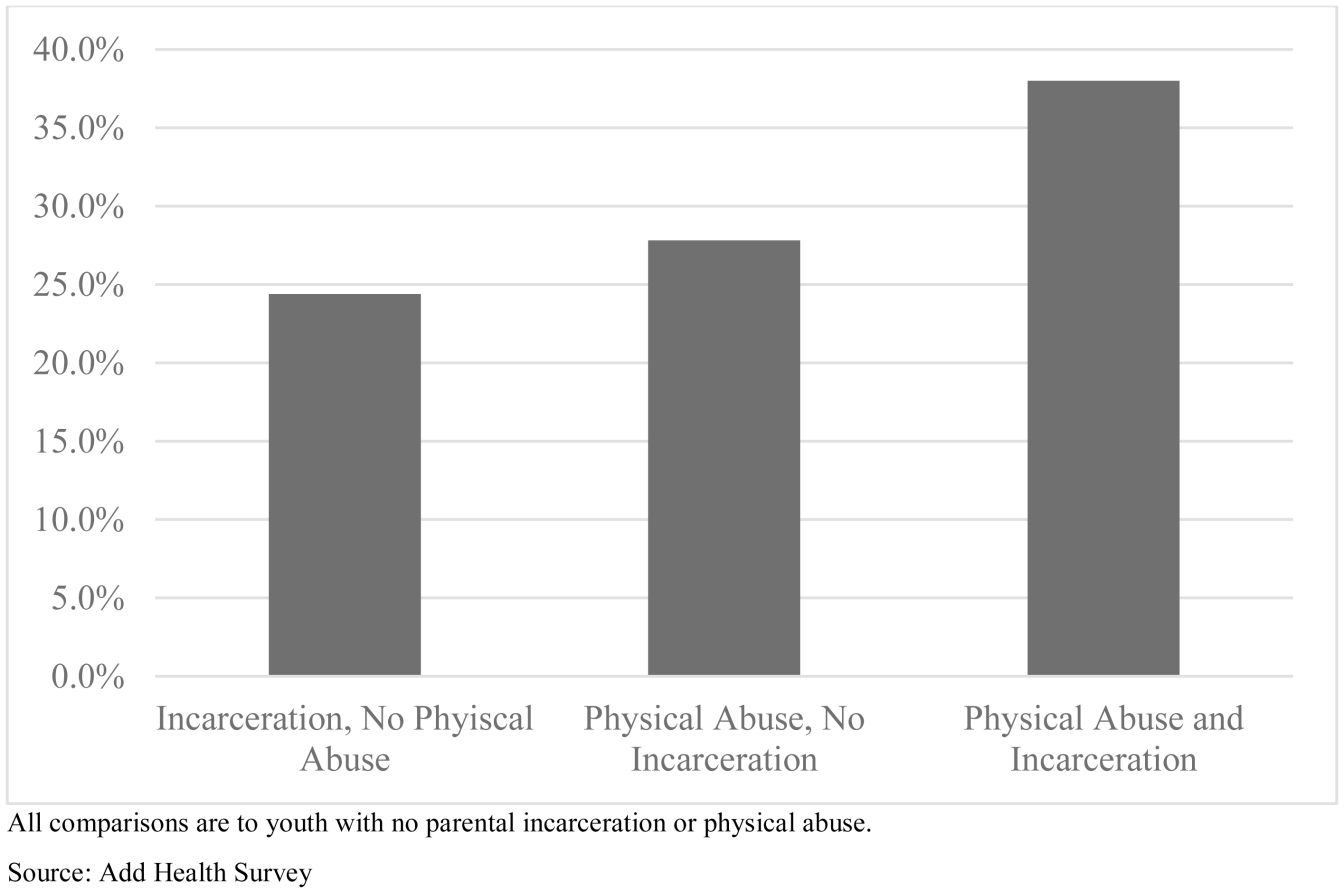
Increases in Delinquency by Paternal Incarceration and History of Physical Abuse

A comparison across the models for the full sample, boys, and girls suggests a similar pattern of relationships between delinquency, paternal incarceration, and reports of abuse. In the full sample, reports of either repeated physical abuse or sexual abuse are associated with higher delinquency. A paternal incarceration between birth and Wave I is also significantly associated with higher delinquency. The interaction term for the co-occurrence of repeated physical abuse and paternal incarceration is statistically significant and negative. This relationship is plotted in Figure 1. As the figure illustrates, it is not the case that a history of physical abuse negates the effect of paternal incarceration, but rather that the two events are not independent or purely additive in their consequences. Paternal incarceration in the absence of physical abuse is associated with 24.4% higher delinquency than youth without either an incarcerated parent or physical abuse. Repeated physical abuse in the absence of paternal incarceration is associated with 27.8% higher delinquency than other youth. Worse off are youth who both have an incarcerated father and report physical abuse, with 38.5% higher delinquency than youth without either risk factor.

**Figure 2:**
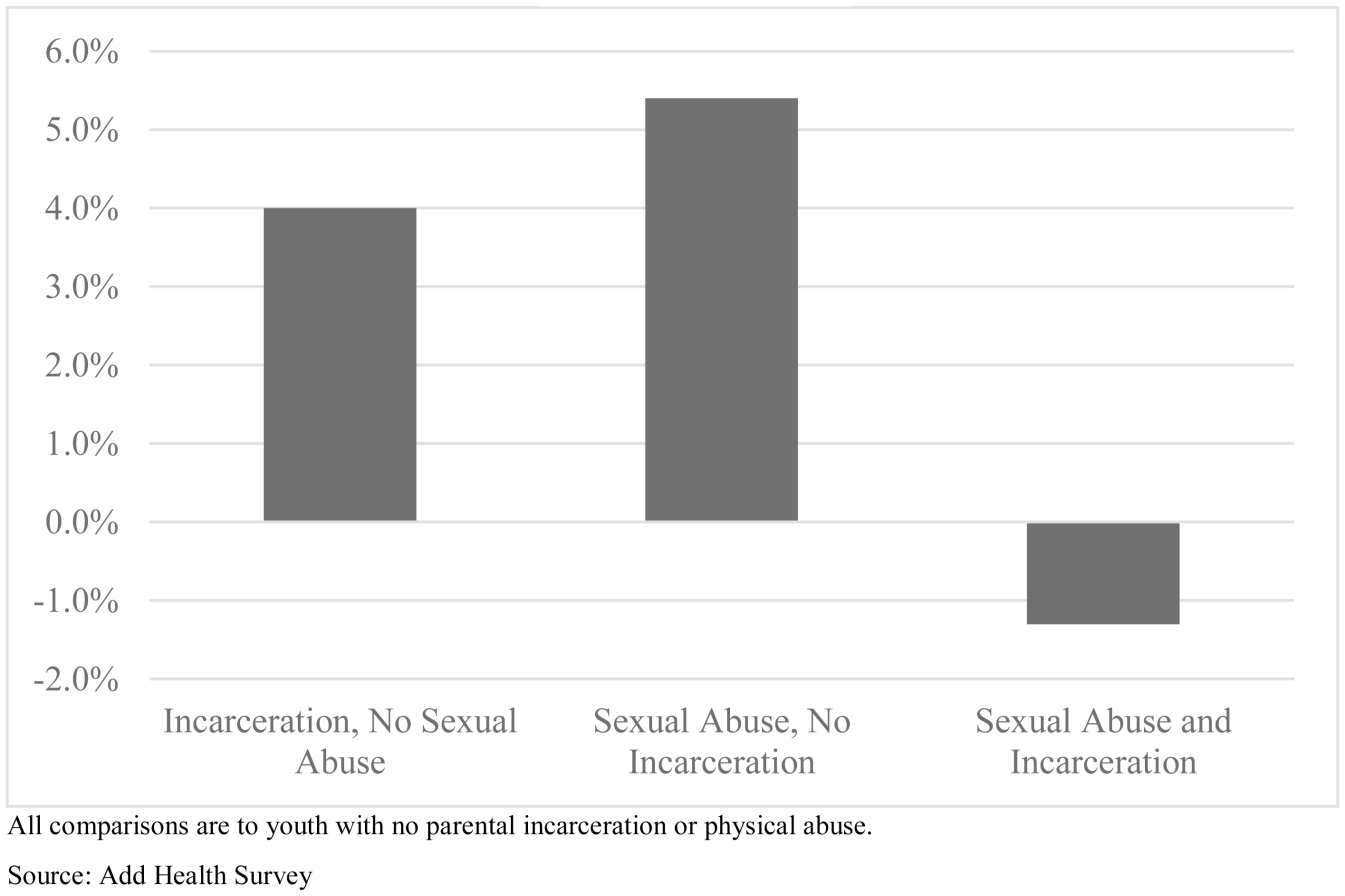
Change in Girls' Depression by Paternal Incarceration and Sexual Abuse in the Family

With respect to depression, an interactive relationship is observed between paternal incarceration and reports of sexual abuse. Interactions of physical abuse and paternal incarceration are not statistically significant. Though present among both boys and girls, and thus in the full sample models, we focus our attention on girls.^51^ We again graph the interaction in Figure 2 for ease of interpretation. As illustrated, paternal incarceration in the absence of sexual abuse in the family is associated with 4.0% higher depression for females. Reports of sexual abuse in the absence of paternal incarceration are associated with 5.4% higher depression. However, paternal incarceration coupled with sexual abuse in the family is associated with 1.3% lower depression (not statistically different from zero) than among girls not reporting either risk factor.

### E. Moderation by Coresidence With Fathers

**Figure 3:**
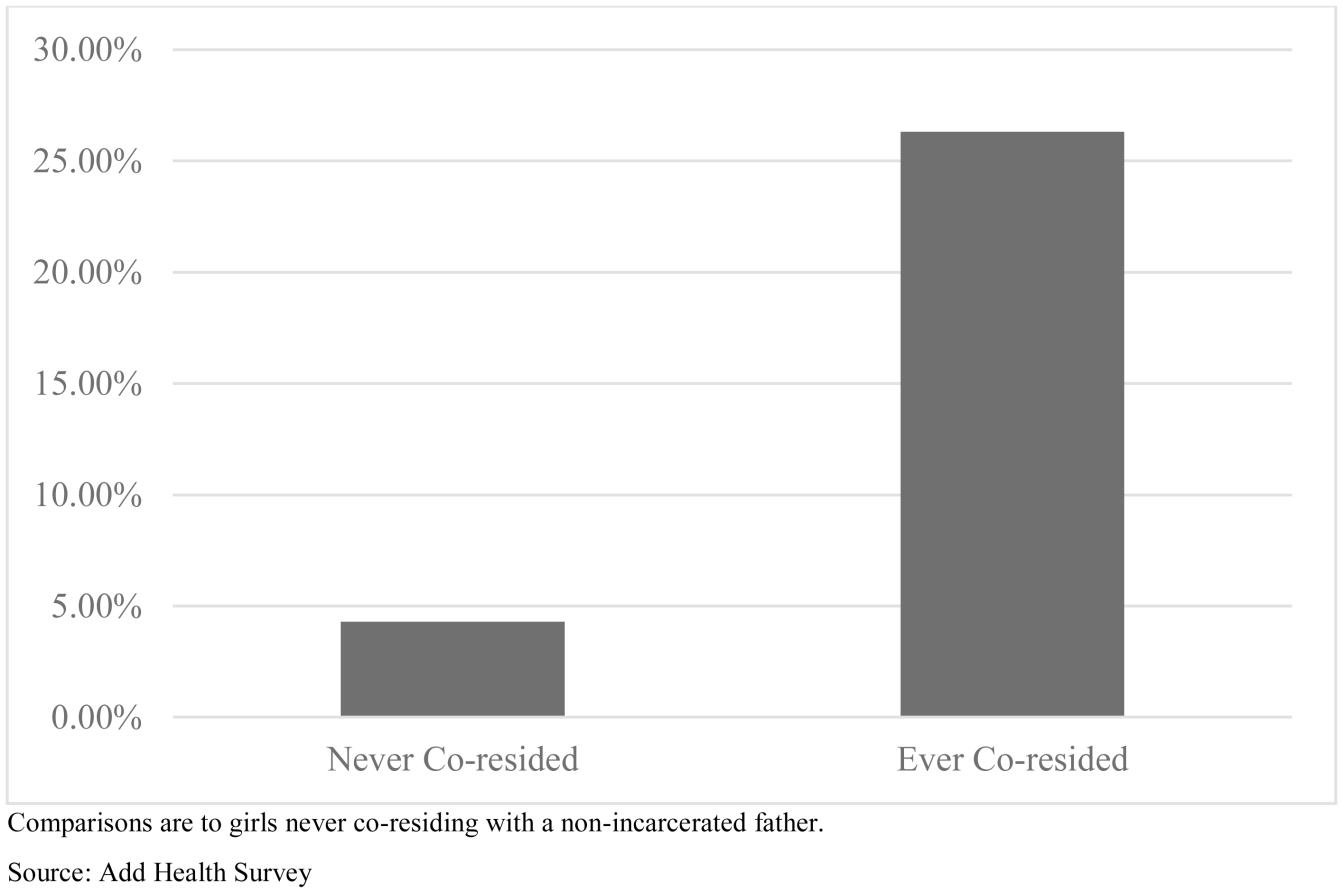
Increases in Girls' Delinquency by Coresidence with an Incarcerated Father

Our last set of analyses considers how associations of paternal incarceration with each outcome might be moderated by whether respondents had ever lived with the father prior to Wave I. These models are presented in Table 5. A rather striking gender-specific pattern is revealed. For boys, associations between paternal incarceration and delinquency do not vary by whether they ever lived with the father, as the interaction term is non-statistically significant. For girls, in contrast, the positive association of paternal incarceration and delinquency is only observed for girls that had ever lived with the father. Figure 3 displays this relationship. Girls who had an incarcerated father with whom they had never lived have the same delinquency as girls without an incarcerated father. Girls who had ever lived with an incarcerated father, in contrast, have 26.3% higher delinquency than girls without an incarcerated father.

**Figure 4:**
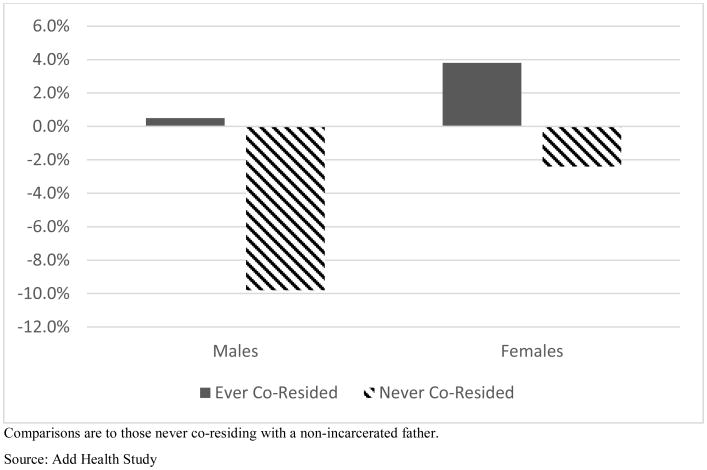
Changes in Depression by Paternal Incarceration and History of Coresidence

Coresidence with the father is also found to moderate associations of paternal incarceration with depression, though in slightly different ways for boys and girls. These relationships are graphed in Figure 4. Among boys, having never lived with an incarcerated father is associated with 9.8% less depression than other boys whose fathers were not incarcerated. Boys who lived with an incarcerated father have essentially the same depression levels as those who never lived with an unincarcerated father. Among girls, having lived with an incarcerated father is associated with 3.8% higher depression than girls who never lived with an unincarcerated father.^52^

## IV. Discussion

This Article contributes to the growing evidence regarding the negative collateral consequences of parental incarceration for youth well-being. In particular, the analyses show that paternal incarceration is consistently associated with adolescent delinquency, but is more tenuously associated with adolescent depression, controlling for measures of family background. Moreover, using a life course perspective, this Article focuses on how these associations are moderated by issues of timing, duration, and frequency of paternal incarceration, as well as by retrospective reports of physical and sexual abuse in the family, and coresidence with the father.

That paternal incarceration is positively associated with adolescent delinquency is consistent with prior research showing paternal incarceration to be strongly associated with aggressive behavior in both childhood and adolescence.^53^ Though this analysis did not employ some of the techniques others have used to assess issues of selection and causality (e.g., fixed effects, propensity score models), several additional findings are consistent with such an interpretation. First, the placebo variable representing paternal incarcerations later in life (i.e., after the outcomes were measured) was unassociated with adolescent delinquency, suggesting that unmeasured factors that may have led to paternal future incarcerations were not driving the associations observed. Secondly, incarcerations at virtually any other time prior to Wave I were significantly associated with delinquency, with rather sizable effect sizes. Lastly, from a “dosage” perspective, longer duration experiences and more frequent incarcerations had stronger associations with delinquency than did shorter or less frequent incarcerations.^54^ The more tenuous association of paternal incarceration with adolescent depression also resonates with past research on the effect of parental incarceration on children's depression.^55^ Suggestive of unobserved factors is the finding that paternal incarcerations measured after the dependent variable are mostly strongly associated with depression.

Nevertheless, several other studies have observed positive associations between paternal incarceration and youth internalizing outcomes.^56^ We were motivated, in part, to delve further into the conditions under which associations between paternal incarceration and adolescent well-being were strongest by this inconsistency of findings with respect to internalizing outcome. Of perhaps most significance are findings with respect to the moderating role of physical and sexual abuse in the family during childhood. Repeated physical abuse during childhood was found to slightly moderate associations of paternal incarceration with delinquency, though youth experiencing both paternal incarceration and reported physical abuse were most disadvantaged. Reports of sexual abuse more clearly moderated the associations of paternal incarceration and depression, particularly for girls. In the absence of sexual abuse in childhood, paternal incarceration was associated with higher depression among girls (though not for boys). In contrast, among girls reporting sexual abuse during childhood, paternal incarceration had no association with depression. This is suggestive of a potentially protective role of paternal incarceration in cases of abuse. At the same time, it is only suggestive, given the fact that the wording of the questions does not specify which parent or adult guardian was involved. Further research is needed to further identify conditions under which parental incarcerations are protective.

Finally, our consideration of moderation by having ever coresided with the father revealed several interesting gender differences. Among girls, paternal incarceration was only found to be significantly associated with delinquency and depression if the respondent had ever lived with the father. Among boys, it depended on the outcome. For delinquency, it did not appear to matter for boys whether they had ever lived with their father, as paternal incarceration was associated with significantly higher delinquency in either case. For depression, in contrast, boys who had not lived with an incarcerated father actually had lower levels of depression than did boys who had not lived with an unincarcerated father. Duration and frequency patterns also point to gender differences in the effects of paternal incarceration. Boys appear to be more responsive to one-time and short duration incarcerations of their fathers, whereas associations were strongest among girls experiencing more frequent or longer incarcerations. Though our interpretation is necessarily speculative, it may be that one-time and short incarcerations (which are likely to be for less serious offenses) are more disruptive to boys who identified with their previously unincarcerated fathers. In contrast, boys may be more likely to write off and not identify with fathers with repeated or more serious offenses.

Future research should further examine such gender differences and the role of past relationships (both good and bad) with the incarcerated father. Relatedly, this research should also consider the interactive relationship between the genders of both the adolescent and their incarcerated parents. This analysis focused on paternal incarceration. In sensitivity analyses, we added an indicator for whether the biological mother had ever been incarcerated. Maternal incarceration was significantly and positively associated with both outcomes,^57^ but the cell sizes were much too small to examine the moderating analyses considered here.

This study has several limitations that should be taken into consideration when interpreting the results. Although the Add Health project offers a nationally representative sample and longitudinal design, its original design was not focused on issues of crime or involvement with the criminal justice system. Questions regarding paternal incarceration were not asked until Wave III, and it was not until Wave IV that the detailed questions used in this analysis were included. Thus, the reports of paternal incarceration are retrospective and likely contain substantial errors of recall. Recall error is likely larger for the questions regarding the ages at which fathers were first incarcerated and last released. We hope that our use of rather broad timing ranges (e.g., before birth, birth to age six, etc.) minimize such error. Measures of duration were also necessarily somewhat crude and likely mask many dynamics occurring between the first incarceration and last release.

Another limitation of the Add Health data is a lack of information regarding paternal criminality. Thus, we cannot be sure that the associations observed here are due to the incarceration itself or the fathers' behaviors that led to incarceration. Our use of placebo measures of paternal incarceration suggest that this may be more of an issue for depression than delinquency, but future research using Add Health might use techniques such as propensity score models to further examine the issue. Finally, as a school-based sample, Add Health likely misses some of the most disadvantaged youth who had already dropped out of the school system.^58^

## Conclusion

This Article finds paternal incarcerations to be a significant risk factor for adolescent delinquency. Results for depression appear to be more contingent on the adolescent's gender and other moderating factors. Concerns regarding causality warrant caution against drawing specific policy recommendations. Nevertheless, we suggest several general considerations. First, given the consistency of the findings regarding adolescent delinquency in this study and in the literature more broadly, delinquency and violence prevention should be an important component of programs designed to assist youth with incarcerated parents. Second, this and other studies warrant such programs considering the offenses for which fathers (and mothers) were incarcerated. Finally, the complexity and contingency of results to issues of past coresidency, and issues of timing and duration of incarcerations, suggest that a one-size-fits-all approach to intervention will be ineffective.

## Figures and Tables

**Table 1:** Weighted Means and Proportions

	Full Sample		Father Incarceration			
	(N=14579)		Yes (N=2089)		No (N=12490)	
Depression (logged)	0.328	(12.91)	0.363	(13.19)	0.323	(34.49)
Delinquency (logged)	1.235	(34.77)	1.442	(35.42)	1.201	(70.01)
Paternal Incarceration (FI)	0.141	(13.42)	1.000	(0.00)	0.000	(9.81)
Don't Know FI	0.060	(9.13)	0.000	(0.00)	0.070	(6.25)
Mother's Incarceration	0.038	(7.36)	0.104	(11.67)	0.027	(0.00)
**Timing of Paternal Incarceration**						
FI Before Birth	0.008	(3.35)	0.054	(8.64)	—	—
FI Release After Birth	0.005	(2.73)	0.036	(7.11)	—	—
FI Ages 0–5	0.038	(7.39)	0.270	(17.00)	—	—
FI Ages 6–12	0.061	(9.23)	0.433	(18.96)	—	—
FI Adolescence	0.008	(3.52)	0.059	(9.05)	—	—
FI Later in Life	0.021	(5.47)	0.146	(13.05)	—	—
**Frequency of Incarceration**						
FI 1 Time	0.060	(9.13)	0.423	(18.91)	—	—
FI 2–3 Times	0.029	(6.50)	0.207	(15.51)	—	—
FI 4+ times	0.021	(5.55)	0.150	(13.67)	—	—
Don't Know Frequency	0.031	(6.68)	0.220	(15.51)	—	—
**Duration of Incarceration**						
FI Duration: 0–1	0.050	(8.39)	0.353	(18.29)	—	—
FI Duration: 2–4	0.012	(4.16)	0.083	(10.58)	—	—
FI Duration: 5–9	0.010	(3.80)	0.070	(9.74)	—	—
FI Duration: 10+	0.020	(5.43)	0.143	(13.41)	—	—
Duration Not Determined	0.030	(6.57)	0.212	(15.65)	—	—
**Demographic Controls**						
Age at Wave I	15.525	(69.82)	15.396	(68.46)	15.55	(19.28)
Girl	0.494	(19.26)	0.486	(19.13)	0.495	(13.58)
Black	0.157	(14.01)	0.228	(16.07)	0.145	(12.35)
Hispanic	0.118	(12.45)	0.133	(13.00)	0.116	(7.01)
Asian	0.031	(6.71)	0.014	(4.45)	0.034	(4.60)
Other Race	0.016	(4.83)	0.025	(6.02)	0.014	(8.75)
Foreign Born	0.052	(8.56)	0.038	(7.28)	0.055	(18.85)
Biological Parents	0.561	(19.12)	0.290	(17.37)	0.605	(13.37)
Step Parents	0.156	(13.97)	0.253	(16.63)	0.140	(14.71)
Single Mother	0.198	(15.35)	0.327	(17.95)	0.177	(6.31)
Single Father	0.028	(6.39)	0.033	(6.82)	0.028	(8.25)
Other Parents	0.055	(8.79)	0.095	(11.23)	0.048	(1.93)
Live Alone	0.003	(1.93)	0.002	(1.89)	0.003	(115.17)
Parent's Education	13.820	(114.02)	12.788	(98.09)	13.989	(13.22)
**Moderators**						
Physical/Sexual Abuse	0.159	(14.08)	0.296	(17.48)	0.136	(7.85)
Sexual Abuse	0.050	(8.42)	0.092	(11.09)	0.043	(12.05)
Physical Abuse	0.128	(12.88)	0.241	(16.37)	0.110	(11.33)
Ever Lived with Father	0.895	(11.85)	0.830	(14.44)	0.905	(0.00)

**Table 2:** Timing of Paternal Incarceration

	Delinquency			Depression		

	Full	Boys	Girls	Full	Boys	Girls
Intercept	1.018‡ (.08)	0.854‡ (.12)	0.978‡ (.10)	-0.016 (.03)	-0.006 (.04)	0.097* (.04)
**Paternal Incarceration**	—	—	—	—	—	—
**Before Birth**	0.207† (.08)	0.248ˆ (.13)	0.164ˆ (.11)	-0.007 (.03)	0.015 (.04)	-0.026 (.04)
**Release After Birth**	0.477‡ (.10)	0.449‡ (.14)	0.525† (.17)	0.046 (.04)	0.057 (.04)	0.021 (.07)
**Ages 0–5**	0.293‡ (.04)	0.286‡ (.06)	0.293‡ (.05)	0.020 (.01)	0.024 (.02)	0.014 (.02)
**Ages 6–12**	0.180‡ (.03)	0.154‡ (.05)	0.199‡ (.04)	0.001 (.01)	-0.030ˆ (.02)	0.035* (.02)
**Adolescence**	0.224 † (.08)	0.132 (.12)	0.302† (.11)	0.048 (.03)	0.033 (.04)	0.063 (.04)
**Later in Life**	0.071 (.05)	0.010 (.08)	0.155* (.07)	0.064‡ (.02)	0.050* (0.02)	0.078† (.03)
Age	0.009* (.00)	0.018† (.01)	-0.001 (.01)	0.022‡ (.00)	0.022‡ (.00)	0.022‡ (.00)
Girl	-0.207‡ (.01)	— —	— —	0.117‡ (.01)	— —	— —
Black	-0.023 (.02)	-0.125‡ (.03)	0.076† (.03)	0.017* (.01)	0.009 (.01)	0.024* (.01)
Hispanic	0.203‡ (.03)	0.196‡ (.04)	0.213‡ (.03)	0.013 (.01)	0.000 (.01)	0.026* (.01)
Asian	0.183‡ (.05)	0.127ˆ (.07)	0.247‡ (.06)	0.069‡ (.02)	0.071‡ (.02)	0.063† (.03)
Other Race	0.137* (.06)	0.250† (.08)	-0.031 (.08)	0.043* (.02)	0.069† (.03)	0.006 (.03)
Foreign Born	-0.240‡ (.04)	-0.329‡ (.06)	-0.143† (.05)	0.017 (.01)	0.017 (.02)	0.019 (.02)
Step Parents	0.121‡ (.02)	0.143‡ (.03)	0.105‡ (.03)	0.044‡ (.01)	0.038‡ (.01)	0.050‡ (.01)
Single Mother	0.111‡ (.02)	0.148‡ (.03)	0.077† (.03)	0.049‡ (.01)	0.035‡ (.01)	0.063‡ (.01)
Single Father	0.295‡ (.04)	0.334‡ (.06)	0.221‡ (.07)	0.135‡ (.02)	0.123‡ (.02)	0.155‡ (.03)
Other Parents	0.058ˆ (.03)	0.067 (.05)	0.068ˆ (.04)	0.071‡ (.01)	0.056‡ (.02)	0.086‡ (.02)
Lived Alone	-0.161 (.15)	-0.336ˆ (.18)	0.614* (.31)	0.034 (.05)	-0.035 (.06)	0.306* (.13)
Parent's Education	0.006* (.00)	0.009* (.00)	0.004 (.11)	-0.007‡ (.00)	-0.007‡ (.00)	-0.007‡ (.00)
R2	0.04	0.03	0.03	0.06	0.04	0.04
F	27.84‡	10.75‡	9.98‡	52.73‡	15.71‡	17.36‡
N	14579	6826	7753	14579	6826	7753

ˆ p< .1

* p< .05

† p< .01

‡ p< .001 (two-sided tests)

**Table 3:** Duration and Frequency of Paternal Incarceration

	Delinquency +			Depression +		
	
	Full	Boys	Girls	Full	Boys	Girls
**Intercept**	1.020‡ (.08)	0.849‡ (.12)	0.983‡ (.10)	-0.017 (.03)	-0.013 (.04)	0.096* (.04)
**Paternal Duration**						
0–1 Year	0.151‡ (.03)	0.102* (.05)	0.202‡ (.05)	0.038‡ (.01)	0.041† (.02)	0.031ˆ (.02)
2–4 Years	0.078 (.07)	0.038 (.10)	0.090 (.09)	-0.040ˆ (.03)	-0.050ˆ (.03)	-0.032 (.040
5–9 Years	0.276‡ (.08)	0.138 (.12)	0.396‡ (.10)	0.029 (.03)	-0.030 (.04)	0.085* (.04)
10+ Years	0.367‡ (.05)	0.298‡ (.08)	0.420‡ (.07)	0.041* (.02)	0.000 (.03)	0.076† (.03)
Don't Know Duration	0.232‡ (.04)	0.234‡ (.07)	0.238‡ (.06)	-0.008 (.02)	-0.029 (.02)	0.016 (.02)
Duration Before Birth	0.344‡ (.07)	0.362‡ (.10)	0.299‡ (.09)	0.013 (.02)	0.036 (.03)	-0.015 (.04)
Still in Prison	-0.236* (.10)	-0.123 (.14)	-0.367† (.14)	0.009 (.04)	-0.010 (.05)	0.038 (.06)
**R2**	0.04	0.03	0.03	0.06	0.04	0.04
**F**	26.65‡	9.88‡	10.46‡	50.19‡	14.93	16.67‡

**Intercept**	1.014‡ (.08)	0.844‡ (.12)	0.977‡ (.10)	-0.015 (.03)	-0.008 (.04)	0.095* (.04)
**Paternal Frequency**	—	—	—	—	—	—
1 Time	0.135‡ (.03)	0.122† (.05)	0.140‡ (.04)	0.016 (.01)	0.030* (.02)	-0.006 (.02)
2–3 Times	0.189‡ (.04)	0.158* (.07)	0.224‡ (.06)	0.002 (.02)	-0.040ˆ (.02)	0.047* (.02)
4+ Times	0.359‡ (.05)	0.234† (.08)	0.476‡ (.07)	0.032ˆ (.02)	0.009 (.03)	0.056* (.03)
Don't Know Frequency	0.278‡ (.04)	0.313‡ (.07)	0.255‡ (.06)	0.038* (.02)	0.003 (.02)	0.071† (.02)
**R2**	0.04	0.03	0.02	0.06	0.04	0.04
**F**	31.06‡	11.67‡	12.04‡	58.49‡	17.43‡	19.79‡
**N**	14575	6826	7753	14575	6826	7753

ˆ p< .1

* p< .05

† p< .01

‡ p< .001 (two-sided tests)

+ Models include controls for Age, Female, Black, Hispanic, Asian, Other Races, Foreign Born, Step Parents, Single Mother, Single Father, Other Parents, Lived Alone, and Parent's Education.

**Table 4:** Paternal Incarceration and Reports of Abuse

	Delinquency+			Depression+		

	Full	Boys	Girls	Full	Boys	Girls
**Intercept**	0.958‡ (.08)	0.815‡ (.12)	0.894‡ (.10)	-0.032 (.03)	-0.012 (.04)	0.063ˆ (.04)
**Paternal Incarceration**	0.244‡ (.03)	0.221‡ (.04)	0.260‡ (.04)	0.011 (.01)f	-0.013 (.01)	0.034* (.01)
**Physical Abuse**	0.278‡ (.02)	0.269‡ (.04)	0.299‡ (.03)	0.063‡ (.01)	0.054‡ (.01)	0.073‡ (.01)
**Paternal Incarceration & Physical Abuse**	-0.136* (.06)	-0.152ˆ (.09)	-0.121ˆ (.08)	-0.016 (.02)	0.020 (.03)	-0.055ˆ (.03)
**R2**	0.04	0.03	0.04	0.07	0.04	0.04
**F**	36.17‡	13.63‡	16.62‡	58.46‡	17.61‡	20.99‡

**Intercept**	0.988‡ (.08)	0.836‡ (.12)	0.937‡ (.10)	-0.028 (.03)	-0.014 (.04)	0.071ˆ (.04)
**Paternal Incarceration**	0.229‡ (.03)	0.203‡ (.04)	0.251‡ (.03)	0.021* (.01)	0.003 (.01)	0.040† (.01)
**Sexual Abuse**	0.102† (.04)	0.084 (.08)	0.116† (.04)	0.080‡ (.01)	0.152‡ (.03)	0.054‡ (.02)
**Paternal Incarceration & Sexual Abuse**	0.032 (.08)	0.217 (.17)	-0.048 (.09)	-0.103‡ (.03)	-0.142† (.06)	-0.107‡ (.04)
**R2**	0.03	0.03	0.02	0.07	0.04	0.04
**F**	28.73‡	10.9‡	11.15‡	57.58‡	18.04‡	19.85‡
**N**	14579	6826	7753	14579	6826	7753

ˆ p< .1

* p< .05

† p<.01

‡ p<.001 (two-sided tests)

+ Models include controls for Age, Female, Black, Hispanic, Asian, Other Races, Foreign Born, Step Parents, Single Mother, Single Father, Other Parents, Lived Alone, and Parent's Education.

**Table 5:** Paternal Incarceration and Coresidence

	Delinquency+			Depression+		

	Full	Boys	Girls	Full	Boys	Girls
**Intercept**	0.967‡ (.08)	0.704‡ (.13)	1.012‡ (.11)	0.004 (.03)	0.027 (.04)	0.098* (.04)
**Paternal Incarceration**	0.148† (.06)	0.275† (.10)	0.043 (.07)	-0.060† (.02)	-0.098† (.03)	-0.024 (.03)
**Ever Lived with Father**	0.009 (.03)	0.058 (.05)	-0.047 (.04)	-0.023* (.01)	-0.036* (.02)	-0.012 (.02)
**Paternal Incarceration & Ever Lived with Father**	0.109ˆ (.07)	-0.068 (.10)	0.266‡ (.08)	0.095‡ (.02)	0.121‡ (.03)	0.074* (.03)
**R2**	0.04	0.03	0.02	0.06	0.04	0.04
**F**	29.63‡	11.84‡	10.67‡	52.00‡	15.97‡	17.11‡
**N**	14579	6826	7753	14579	6826	7753

ˆ p< .1

* p< .05

† p< .01

‡ p< .001 (two-sided tests)

+ Models include controls for Age, Female, Black, Hispanic, Asian, Other Races, Foreign Born, Step Parents, Single Mother, Single Father, Other Parents, Lived Alone, and Parent's Education.

## Footnotes

1 This research uses data from the National Longitudinal Study of Adolescent Health (Add Health), a program project directed by Kathleen Mullan Harris and designed by J. Richard Udry, Peter S. Bearman, and Kathleen Mullan Harris at the University of North Carolina at Chapel Hill, and funded by grant P01-HD31921 from the Eunice Kennedy Shriver National Institute of Child Health and Human Development, with cooperative funding from twenty-three other federal agencies and foundations. Special acknowledgment is due to Ronald R. Rindfuss and Barbara Entwisle for assistance in the original design. Information on how to obtain the Add Health data files is available on the Add Health website (http://www.cpc.unc.edu/addhealth, *archived at* http://perma.cc/4CLK-2BG8). No direct support was received from grant P01-HD31921 for this analysis.

2 *See, e.g.,* Mass Imprisonment: Social Causes and Consequences (David Garland ed., 2001) (using the term “mass incarceration” to denote both a high rate of incarceration and a disproportionate impact of incarceration on minority groups); Stephen Raphael & Michael A. Stoll, Why Are So Many Americans in Prison? (2013) (documenting high rates of incarceration in the United States); Christopher Wildeman, *Parental Imprisonment, the Prison Boom, and the Concentration of Childhood Disadvantage*, 46 Demography 265 (2009) (estimating the cumulative risk of having an incarcerated parent by age fourteen for demographic subgroups). *See also* Becky Pettit & Bruce Western, *Mass Imprisonment and the Life Course: Race and Class Inequality in U.S. Incarceration*, 69 Am. Soc. Rev. 151, 162 (2004).

3 *See* Jeff Manza & Christopher Uggen, Locked Out: Felon Disenfranchisement and American Democracy (2006) (documenting changes in state laws regarding the right of felons to vote, and how these changes likely affected election outcomes); Devah Pager, Marked: Race, Crime, and Finding Work in an Era of Mass Incarceration (2007) (conducting experimental study documenting the accentuated disadvantage of a prison record for African-American job applicants); Bruce Western, Punishment and Inequality in America (2006) (examining consequences of parental incarceration for labor market outcomes and family and child well-being); Michael Massoglia & Jason Schnittker, *No Real Release*, 8 Contexts 38 (2009) (discussing the negative health consequences of parental incarceration for both parents and children).

4 *See, e.g.,* Donald Braman, Doing Time on the Outside: Incarceration and Family Life in Urban America (2004) (discussing the potential consequences of parental incarceration on families and children); Megan Comfort, Doing Time Together: Love and Family in the Shadow of the Prison (2007) (reviewing effects of parental incarceration on children and families); Jeremy Travis & Michelle Waul, Prisoners Once Removed: The Impact of Incarceration and Reentry on Children, Families, and Communities (2003) (providing a discussion of parental incarceration and reentry into the community on families and their children); John Hagan & Ronit Dinovitzer, *Collateral Consequences of Imprisonment for Children, Communities, and Prisoners*, 26 Crime & Just. 121 (1999) (discussing the many collateral consequences of parental imprisonment on children and families).

5 *See* Joseph Murray & David P. Farrington, *The Effects of Parental Imprisonment on Children*, 37 Crime & Just. 133 (2008). Murray & Farrington provided a meta-analysis of existing studies and new evidence from the Cambridge Study in Delinquent Development on the associations between parental imprisonment and a wide range of child outcomes, including outcomes related to antisocial behavior, mental health, alcohol and drug use, and educational performance. *Id.*

6 *See* Holly Foster & John Hagan, *Incarceration and Intergenerational Social Exclusion,* 54 Soc. Probs. 399 (2007). Foster & Hagan theorized that paternal incarceration is part of an intergenerational process of social exclusion. *Id.* The authors empirically showed that political disengagement, homelessness, and lack of health insurance among children of the incarcerated during the transition to adulthood, but also demonstrated that these associations are mediated by lower levels of educational attainment. *Id*.

7 *See e.g.,* Amanda Geller et al., *Beyond Absenteeism: Father Incarceration and Child Development*, 49 Demography 49 (2011). Geller et al. found larger associations of parental incarceration, particularly for aggressive behavior, from a comparison of the effects of paternal incarceration on childhood internalizing and externalizing outcomes to those of other paternal separations (e.g., divorce and death). *Id.* The authors used fixed effects models to control for unobserved factors potentially driving the associations. *Id.* They still found a statistically significant relationship with childhood aggressive behavior. *Id. See also* Joseph Murray et al., *Children's Antisocial Behavior, Mental Health, Drug Use, and Educational Performance After Parental Incarceration: A Systematic Review and Meta-analysis*, 138 Psychol. Bull. 175 (2012). Murray et al. focused on rigorous studies with comparison groups or controls for parental criminality or children's prior antisocial behavior. *Id.* They found significant associations between parental incarceration and children's antisocial behavior, but found no relationship with other outcomes such as poor mental health, drug use, or lower educational performance. *Id.* The authors advocated for future research with more rigorous tests of a causal relationship between parental incarceration and child outcomes. *Id. See also* Christopher Wildeman, *Paternal Incarceration and Children's Physically Aggressive Behaviors: Evidence from the Fragile Families and Child Wellbeing Study*, 89 Soc. Forces 285 (2010). Wildeman used a variety of statistical techniques to control for competing arguments and provided strong evidence of a positive association between paternal incarceration and children's physical aggression for boys, but a negative association for girls. *Id.*

8 *See generally* Elizabeth I. Johnson & Beth Easterling, *Understanding Unique Effects of Parental Incarceration on Children: Challenges, Progress, and Recommendations*, 74 J. Marriage & Fam. 342 (2012) (discussing issues related to the many risk factors in the lives of families of incarcerated parents before parental incarceration, which may render associations between parental incarceration and child outcomes spurious, and positing that parental incarceration may be protective of child well-being in cases of parental violence or abuse); Christopher Wildeman et al., *Misidentifying the Effects of Parental Incarceration? A Comment on Johnson and Easterling (2012)*, 75 J. Marriage & Fam'y. 252 (2013) (commenting on and disagreeing with Johnson & Easterling's assertion of increasing evidence of a positive or protective effect of parental incarceration).

9 *See generally* Joseph Murray et al., *Parental Involvement in the Criminal Justice System and the Development of Youth Theft, Marijuana Use, Depression, and Poor Academic Performance*, 50 Criminology 255 (2012) (finding that the association between parental incarceration and theft by boys was not moderated by the timing of incarceration).

10 *See generally* Murray et al., *supra* note 7 (examining gender differences in the effects of parental incarceration).

11 *See* Murray & Farrington, *supra* note 5, at 135.

12 *See* Foster & Hagan, *supra* note 6, at 400; Murray & Farrington, *supra* note 5, at 135; Christopher Wildeman et al., *Despair by Association? The Mental Health of Mothers with Children by Recently Incarcerated Fathers*, 77 Am. Soc. Rev. 216, 219 (2012).

13 *See* Peggy Giordano, Legacies of Crime: A Follow-Up of the Children of Highly Delinquent Girls and Boys 215 (2010); Maureen R. Waller & Raymond Swisher, *Fathers' Risk Factors in Fragile Families: Implications for “Healthy” Relationships and Father Involvement*, 53 Soc. Probs. 392, 399 (2006).

14 *See* Wildeman, *supra* note 7, at 297. *See also* Sarah Wakefield & Christopher Wildeman, *Mass Imprisonment and Racial Disparities in Childhood Behavioral Problems*, 10 Criminology & Pub. Pol'y. 793, 789–99 (2011). The Fragile Families and Child Wellbeing Study began in 1998 and is a longitudinal study of new births in twenty U.S. cities. Most of the births included in the study are to unmarried parents, though a control group of births to married parents is also included. Follow-up interviews were conducted when the children were one, three, five, nine, and fifteen.

15 *See* Geller et al., *supra* note 7, at 62.

16 *See* Murray et al., *supra* note 9, at 272–73. The Pittsburgh Youth Study is a longitudinal study of inner-city boys who were enrolled in public schools and were between the ages seven and thirteen from 1987 to 1988. Follow-up interviews were conducted until the oldest boys were age twenty-four.

17 *See* Geller et al., *supra* note 7, at 65.

18 *See* Murray et al., *supra* note 7, at 190–91.

19 *See* Wakefield & Wildeman, *supra* note 14, at 799.

20 *See generally* Robert Crosnoe & Monica Kirkpatrick Johnson, *Research on Adolescence in the Twenty-First Century*, 37 Ann. Rev. Soc. 439 (2011) (discussing developmental and social issues that are unique to adolescents at the turn of the century).

21 *See* Joseph Murray & David P. Farrington, *Parental Imprisonment: Effects on Boys' Antisocial Behavior and Delinquency Through the Life Course*, 46 J. Child Psychol. & Psychiatry 1269, 1272 (2005).

22 *See* Murray et al., *supra* note 9, at 272–73.

23 *See* Wakefield & Wildeman, *supra* note 14.

24 *See* Michael E. Roettger & Raymond R. Swisher, *Associations of Fathers' History of Incarceration with Sons' Delinquency and Arrest Among Black, White, and Hispanic Males in the United States*, 49 Criminology 1109, 1135 (2011).

25 *See* Michael E. Roettger et al., *Paternal Incarceration and Trajectories of Marijuana and Other Illegal Drug Use from Adolescence into Young Adulthood: Evidence from Longitudinal Panels of Males and Females in the United States*, 106 Addiction 121, 128 (2010).

26 *See* Joseph Murray & David P. Farrington, *Parental Imprisonment: Long-lasting Effects on Boys' Internalizing Problems Through the Life Course*, 20 Dev. & Psychopathology 273, 281–82 (2008).

27 *See* Wakefield & Wildeman, *supra* note 14, at 799.

28 *See* Raymond R. Swisher & Michael E. Roettger, *Father's Incarceration and Youth Delinquency and Depression: Examining Differences by Race and Ethnicity*, 22 J. Res. On Adolescence 597, 601 (2012).

29 *See* Murray et al., *supra* note 9, at 273.

30 *See* Susan D. Phillips et al., *Parental Incarceration Among Adolescents Receiving Mental Health Services*, 11 J. Child & Fam. Stud. 385, 395–96 (2002).

31 *See* Murray et al., *supra* note 7, at 190–91.

32 *See* Wildeman, *supra* note 7, at 304.

33 *Id*.

34 *See* Geller et al., *supra* note 7, at 66.

35 *Id*.

36 *See* Bureau of Justice Statistics, U.S. Dep't. of Justice, NCJ 222984, Parents in Prison and Their Minor Children 4 (2008).

37 *See* Geller et al., *supra* note 7, at 66.

38 *See* Murray et al., *supra* note 9, at 274.

39 *See* Rosa Minhyo Cho, *Maternal Incarceration and Children's Adolescent Outcomes: Timing and Dosage*, 84 Soc. Serv. Rev. 257, 270–71 (2010).

40 *See* Murray et al., *supra* note 7, at 192 (identifying frequency and duration of parental incarceration as important issues for future research).

41 *See* Cho, *supra* note 39, at 273–74.

42 *See* Kathleen Mullan Harris & S, Philip Morgan, *Fathers, Sons, and Daughters: Differential Paternal Involvement in Parenting*, 53 J. Marr. & Fam. 531, 540 (1991).

43 *See generally* Sara Raley & Suzanne Bianchi, *Sons, Daughters, and Family Processes: Does Gender of Children Matter?*, Ann. Rev. Soc. 401 (2006) (providing a broad review of the literature on differences in family social processes and variations by the genders of the child and parent).

44 *See* Marcia J. Carlson, *Family Structure, Father Involvement, and Adolescent Behavioral Outcomes*, 68 J. Marriage & Fam. 137, 149 (2006).

45 *See* Murray et al., *supra* note 7, at 187.

46 *See* Geller et al., *supra* note 7, at 68; Wildeman, *supra* note 7, at 296–97.

47 *See* Kathleen Mullan Harris & J. Richard Udry, ICPSR21600-v15, National Longitudinal Study of Adolescent Health (Add Health), 1994–2008 (May 14, 2014), http://doi.org/10.3886/ICPSR21600.v15, *archived at* http://perma.cc/TQS7-5SRQ.

48 A weighted comparison to the full Wave I sample indicates that youth in the analytic sample are slightly more likely to have parents with higher educations and to have lived with both biological parents in adolescence.

49 For concision, we use the term “father” throughout this section, but the underlying data only includes information about biological fathers. The term “father” in this Part should therefore be read to include only biological fathers, not fathers by marriage or social relationship.

50 *See* Wildeman, *supra* note 7, at 295; Geller et al., *supra* note 7, at 61.

51 Caution should be exercised when interpreting the co-occurrence of sexual abuse and paternal incarceration for boys, as these results represent only thirty-nine of the youth in our analytical sample. That a similar pattern was observed for girls, however, suggests the results are not likely to be an anomaly.

52 We do not believe these interactions are driven by small cell sizes, as 25% of boys with an incarcerated father (n = 195) had never lived with him at Wave I. Similar percentages are observed for girls.

53 *See* Geller et al., *supra* note 7, at 62; Murray et al., *supra* note 7, at 191; Roettger & Swisher, *supra* note 24, at 1135; Wakefield & Wildeman, *supra* note 14, at 799; Wildeman, *supra* note 7, at 297.

54 We recognize longer durations are also likely a proxy for the seriousness of the offenses that led to paternal incarceration.

55 *See* Geller et al., *supra* note 7, at 65; Murray et al., *supra* note 7, at 190.

56 *See* Murray & Farrington, *supra* note 5, at 157; Swisher & Roettger, *supra* note 28, at 601; Wakefield & Wildeman, *supra* note 14, at 799.

57 Inclusion of a control for the biological mother's incarceration does not change any of the substantive conclusions reported.

58 One way that some Add Health researchers address this issue is through limiting analyses to younger respondents for whom dropout rates in the population are lower. In additional sensitivity analyses (available from the authors upon request), we stratified our full sample models by age (i.e., middle school versus high school at Wave I). The basic pattern of results presented here was consistent across both age groups.

